# Comparative proteogenomic analysis of right-sided colon cancer, left-sided colon cancer and rectal cancer reveals distinct mutational profiles

**DOI:** 10.1186/s12943-018-0923-9

**Published:** 2018-12-21

**Authors:** Robin Imperial, Zaheer Ahmed, Omer M. Toor, Cihat Erdoğan, Ateeq Khaliq, Paul Case, James Case, Kevin Kennedy, Lee S. Cummings, Niklas Melton, Shahzad Raza, Banu Diri, Ramzi Mohammad, Bassel El-Rayes, Timothy Pluard, Arif Hussain, Janakiraman Subramanian, Ashiq Masood

**Affiliations:** 1 Department of Medicine, University of Missouri-Kansas City School of Medicine, Kansas City, MO 64108 USA; 2 Division of Oncology, Saint Luke’s Cancer Institute, University of Missouri School of Medicine, 4321 Washington St, Kansas City, MO 64111 USA; 3 Department of Computer Engineering, Namik Kemal University, Tekirdag, Turkey; 4 ASPIRE Foundation, Saint Luke’s Health System of Kansas City, Kansas City, MO 64111 USA; 5 Division of Cardiovascular Research, Saint Luke’s Hospital, Kansas City, MO 64111 USA; 6 Department of Surgery, University of Missouri-Kansas City, Kansas City, MO 64108 USA; 7 Department of Computer Sciences, Missouri University of Science and Technology, Rolla, MO 65409 USA; 8 Department of Computer Engineering, Yildiz Technical University, Istanbul, Turkey; 9 Wayne State University, Karmanos Cancer Institute, Detroit, MI 48201 USA; 10 Department of Hematology and Medical Oncology, Winship Cancer Institute, Emory University, Atlanta, GA 30322 USA; 11 Division of Oncology, University of Maryland Greenebaum Comprehensive Cancer Center, Baltimore, MD 20201 USA; 12 Baltimore Veterans Affairs Medical Center, Baltimore, MD 21201 USA; 13 Division of Hematology/Oncology and Cell Therapy, Rush University Medical Center, Chicago, IL 60612 USA

**Keywords:** Right-sided colon cancer, Left-sided colon cancer, Rectal cancers, Clonal evolution, Proteomics, Hotspot mutations

## Abstract

Right-sided colon cancer (RCC) has worse prognosis compared to left-sided colon cancer (LCC) and rectal cancer. The reason for this difference in outcomes is not well understood. We performed comparative somatic and proteomic analyses of RCC, LCC and rectal cancers to understand the unique molecular features of each tumor sub-types. Utilizing a novel in silico clonal evolution algorithm, we identified common tumor-initiating events involving APC, KRAS and TP53 genes in RCC, LCC and rectal cancers. However, the individual role-played by each event, their order in tumor development and selection of downstream somatic alterations were distinct in all three anatomical locations. Some similarities were noted between LCC and rectal cancer. Hotspot mutation analysis identified a nonsense mutation, APC R1450* specific to RCC. In addition, we discovered new significantly mutated genes at each tumor location, Further in silico proteomic analysis, developed by our group, found distinct central or hub proteins with unique interactomes among each location. Our study revealed significant differences between RCC, LCC and rectal cancers not only at somatic but also at proteomic level that may have therapeutic relevance in these highly complex and heterogeneous tumors.

**Electronic supplementary material:**

The online version of this article (10.1186/s12943-018-0923-9) contains supplementary material, which is available to authorized users.

Often grouped as one disease, right-sided colon cancer (RCC, originating from cecum, ascending colon, hepatic flexure) and left-sided colon cancer (LCC, originating from splenic flexure, descending colon, sigmoid colon) represent clinically distinct entities with significant differences in their prognosis and treatment outcomes [1, 2]. Therefore, given their anatomic continuity, the reason for these clinical differences presumably lie at the molecular level delineated by embryological origin. Previous studies have sought to identify these differences by analyzing significantly mutated genes and RNA expression [3, 4]. However, molecular differences including significant specific amino acid alterations (hot spots), proteomic differences and order of mutations in clonal evolution of these tumors have not been studied. We used somatic and proteomic data of colorectal cancers from The Cancer Genome Atlas (TCGA) [4, 5], Memorial Sloan Kettering Cancer Center (MSKCC) [6] and The Cancer Proteome Atlas (TCPA) [7] to study proteogenomic differences in these tumors (See Additional files 1 and 2).

## Results and discussion

### Clonal evolution trajectories

**Fig. 1:**
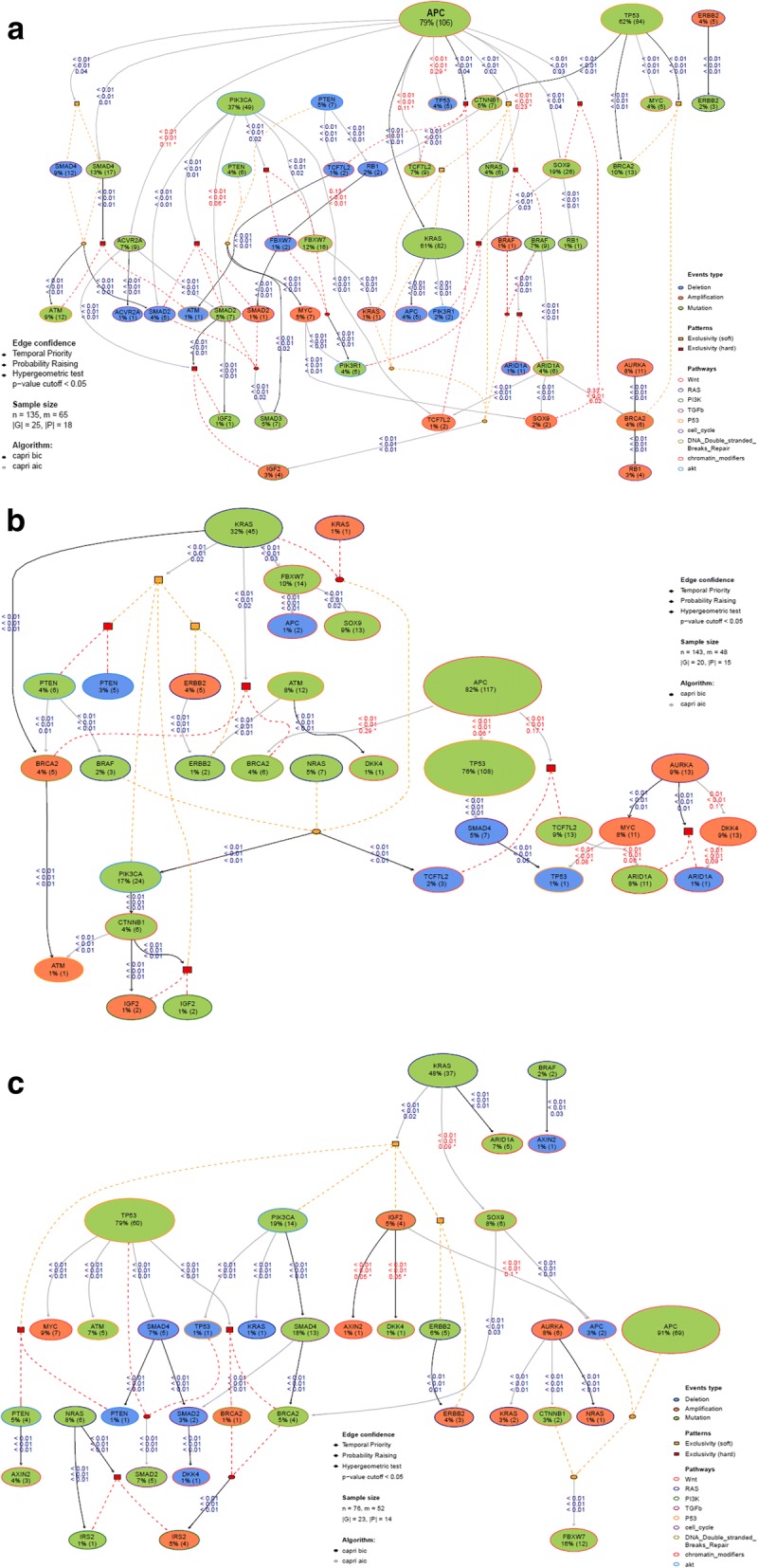
shows ensemble-level clonal evolution trajectories in colorectal cancer using CAPRI algorithm. The events of the model are connected by dashed lines where red dotted lines denote hard and orange denotes soft exclusivity. Algorithm uses both Bayesian information criterion ‘BIC’ and Akaike information criterion ‘AIC’ as a regularization. Non-parametric bootstrap scores (NPB) are shown in the figure with hypergeometric test *p*-value cutoff of < 0.05. Other relations including temporal priority, probability raising are shown in Fig. 1a, b, and c and reported data in Additional file 4. 1a) clonal evolution in RCC, 1b) clonal evolution in LCC and 1c) clonal evolution in rectal cancers

Understanding the mutational timing and evolutionary trajectory of tumors is key to investigate the molecular underpinnings of cancer development and progression. Thus, we applied the PiCnIc (Pipeline for Cancer Inference) algorithm to our data to study ensemble-level cancer progression models and predict the evolutionary mutational trajectories between RCC, LCC and rectal cancers in the TCGA cohort (see Additional file 3). All three cancer locations had mutations in APC, TP53 and KRAS, possibly reflecting common initiating somatic events (Fig. 1). However, there were differences in the hierarchical groupings of mutations that surrounded these events.

In RCC (*n* = 135; Fig. 1a; Additional file 4: Table S1), APC somatic mutations and TP53 somatic mutations were independent events. APC somatic mutations ‘selected’ for KRAS mutations or amplifications. APC somatic mutations also ‘selected’ for SMAD4 somatic mutations or deletions, BRAF mutations and amplification. KRAS and BRAF showed mutual exclusivity. Interestingly, alterations in FBWX7, TCF7L2, and SMAD2 clustered in RCC tumors harboring APC and PIK3CA mutations. With respect to TP53, alterations in this location were associated with CTNNB1, MYC or/and BRCA2 mutations.

In LCC (*n* = 143; Fig. 1b; Additional file 4: Table S2), KRAS somatic mutations ‘selected’ for BRCA2 amplification, PTEN deletions or somatic mutations, PIK3CA somatic mutations, IGF2 amplification or somatic mutations and ERBB2 amplification or somatic mutations. Unlike RCC, alterations in PIK3CA were a late event in LCC and IGF2 amplification via CTNNB1. APC seemed to ‘select’ for TP53, but this did not reach statistical significance (*p* = 0.06). Similarly, APC somatic mutations ‘selected’ for BRCA2 mutations and TCF7L2 somatic mutations or deletions, but this association also did not reach statistical significance (*p* = 0.3 and *p* = 0.2, respectively).

In rectal cancers (*n* = 76; Fig. 1c; Additional file 4: Table S3), key initial mutations are split between TP53 and KRAS. TP53 ‘selects’ for MYC amplification, SMAD4 deletion and BRCA2 somatic mutation or amplification. KRAS ‘selects’ for PTEN deletion or somatic mutations, PIK3CA somatic mutations, IGF2 amplification and ERBB2 amplification or somatic mutations. Among rectal cancer patients with AURKA mutations there is clustering of NRAS amplifications.

Our model shows significant differences in the mutational profiles of genes between RCC and LCC; the early common somatic gene mutations are associated with the ‘selection’ of different subsequent genomic events in RCC compared to LCC. Our results suggest that although LCC and rectal cancers have some similarities in the tumor progression model wherein KRAS ‘selected’ for several genes in common (such as PIK3CA, IGF2, and ERBB2 alterations), significant differences were also noted between these two sites. Taken altogether, our results show non-adherence to the established Vogelstein linear progression model of colorectal cancer progression from normal mucosa to adenoma to carcinoma [8]. Further, our data suggest that RCC, LCC and rectal cancers have distinct mutational behavior in the context of their evolutionary trajectories, mutational timing during cancer development and progression. However, initial events such as mutation in the gatekeeper gene, APC, appear to be similar in colorectal cancers irrespective of location.

### Mutation hotspot analysis

**Fig. 2:**
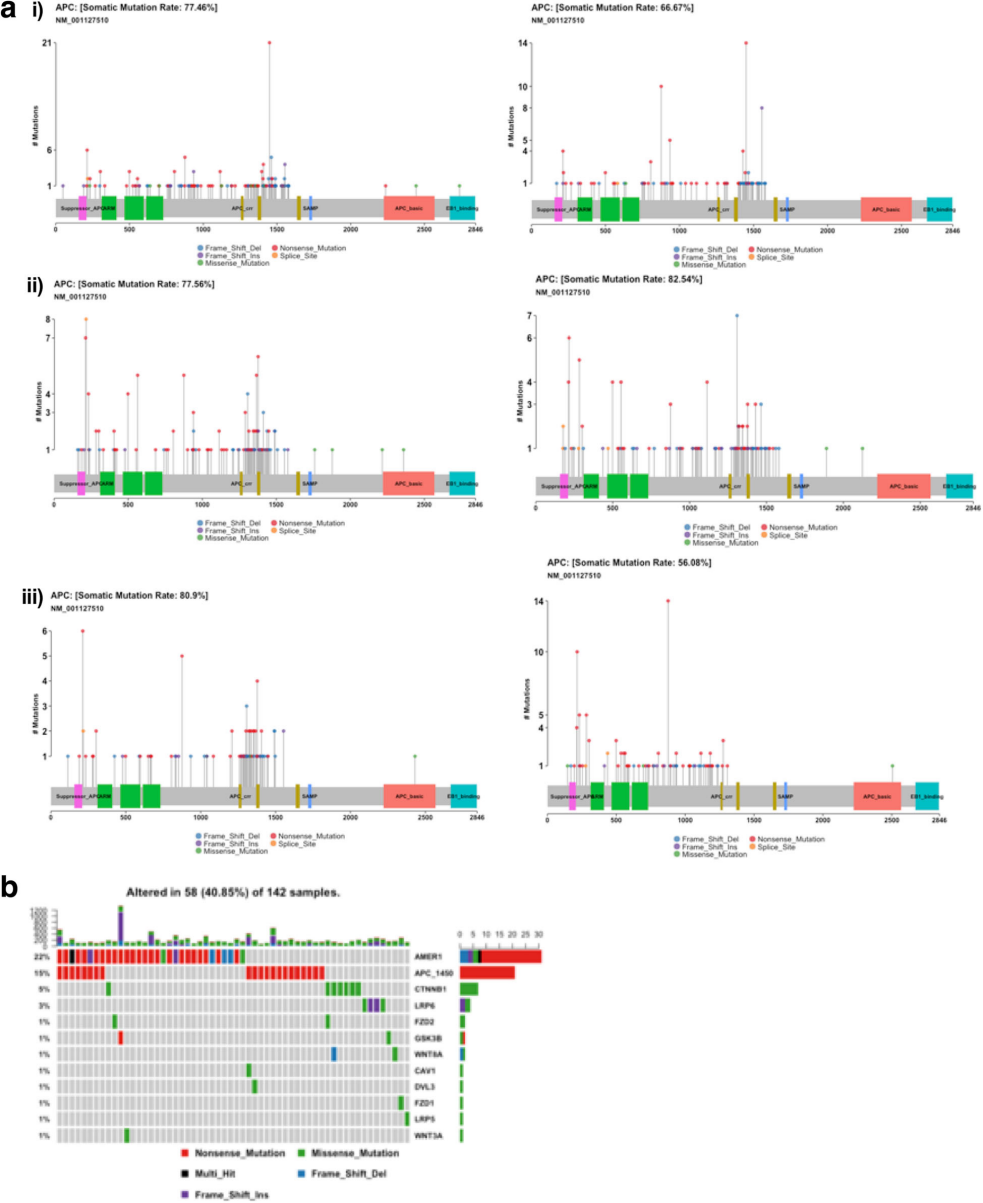
**a** shows the frequency of APC hotspot the R1450 residue in (i) right-sided colon cancers, (ii) left-sided colon cancers and (iii) rectal cancers in TCGA (left) and MSKCC (right) datasets. Y-axis represent total number of mutations at each residue. **b** shows the mutual exclusivity of APC R1450* (APC_1450) compared to other genes of β-Catenin destruction complex in RCC. “APC_MCR” represents other APC mutations within the MCR region that are not at the 1450 residue. The bar plot above the oncoplot represents total mutations in each sample

We studied somatic mutations at the residue sites that can disrupt functional protein domains leading to tumorigenesis and clonal evolution via selective pressure (see Methods in Additional file 3). We found APC R1450* to be a significant mutation specifically enriched in RCC (12–15%) compared to LCC (1%) and rectal tumors (1%) in both the TCGA and MSKCC datasets (all *p* < 0.001, Fig. 2a). To our knowledge, this is the first report to describe the APC R1450* mutation as being predominantly located in RCC. This particular hotspot in APC is exclusively a truncation mutation and lies within the MCR domain (residues 1282–1581; [9]) of the protein, which is a highly mutated area. The resulting truncated mutant conserves beta-catenin binding sites (15 AA repeats) but loses all three axin-binding sites (SAMP repeats) and microtubule interaction via EB1 and PdZ domains. Unlike APC R1450*, the frequency of other mutations within this region is relatively similar among the TCGA and MSKCC data sets. The relative frequencies of non-R1450* mutations within the MCR domain of APC for RCC were 63 and 64% in the TCGA and MSKCC data sets, respectively, for LCC 52 and 51%, respectively, and for rectal cancers 64% vs 58% (which did not meet statistical significance, *p* = 0.35). APC R1450* mutations are mutually exclusive from β-catenin destruction complex genes suggesting that they may be early events in right-sided colon cancer tumorigenesis (Fig. 2b). Given the recent findings by Zhang et al. demonstrating the efficacy of TASIN-1(small molecule inhibitor) in a murine xenograft model of human colorectal cancer harboring a truncation mutation (A1309*) similar to APC R1450* suggests that this mutation may be viable therapeutic target [10]. In addition, we performed significantly mutated gene analysis and discovered newer driver genes at each location (see Additional file 5: Figure S1).

### Proteogenomic analysis

**Fig. 3:**
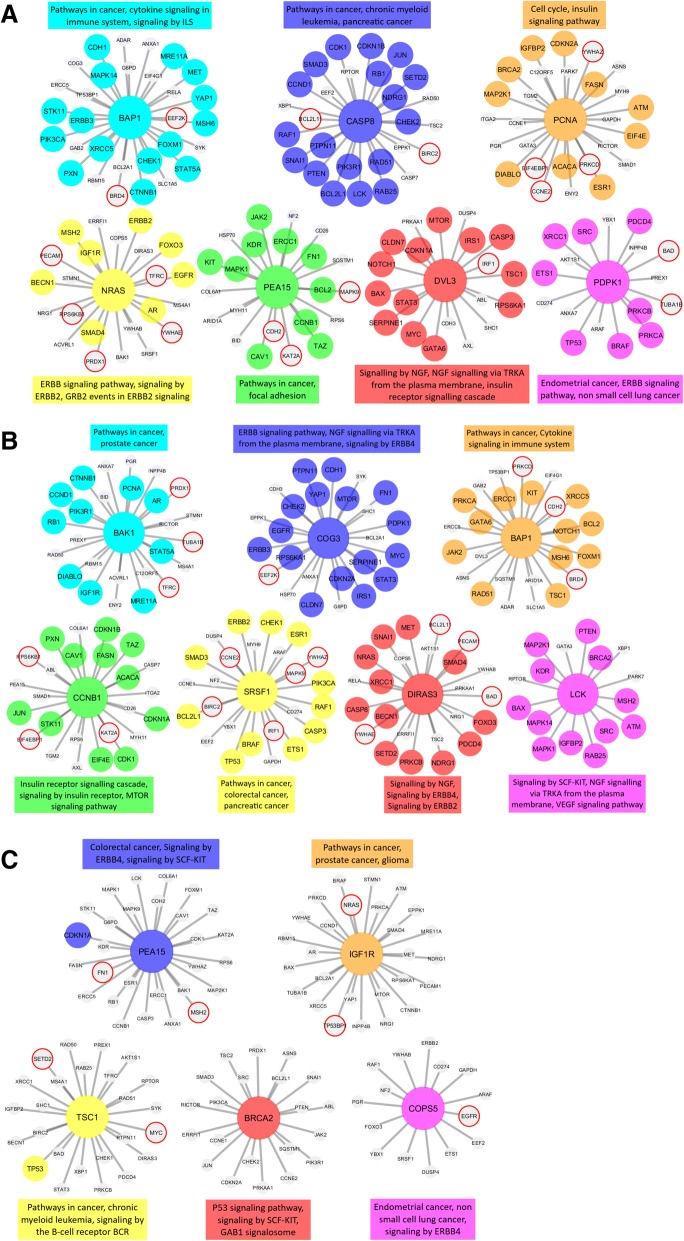
shows the hub genes and neighbors in the disease-related sub-networks obtained by the most successful KDE method (in terms of precision score) in **a** RCC, **b** LCC and **c** rectal cancers. The genes registered in DisGeNET and experimentally confirmed for the diseases are shown with colored and larger nodes. Among these, genes that are not colored but have a red frame have a PMID value of one (e.g. have one supporting publication). There is no entry in DisGeNET for the grey colored nodes. Also, the most associated top three biological pathways, to which each module is related, are given above or below the relevant module to annotate each module

Using The Cancer Proteome Atlas (TCPA) data, we examined RCC, LCC and rectal cancers by proteomic cancer co-expression subnetworks using association estimators methodology previously described by our group (see Methods in Additional file 3). Interestingly, no common protein emerged as having a centralized role (hub protein) across all 3 cancer locations. Within protein-protein interaction networks, several hub proteins, and their respective interactomes, were found to be unique to each of the locations (Fig. 3, see Additional file 6: Table S4).

Several hub proteins that might have a major role in RCC were identified: BAP1 (tumor suppressor gene) CASP8 (apoptosis) PCNA (DNA repair) NRAS (RTK-RAS pathway) PEA15 (apoptosis and RET signaling) DVL3 (cell proliferation and ATM-dependent DNA damage response) and PDPK1 (growth regulation) (Fig. 3a). The potentially significant hub proteins in LCC were: BAP1, BAK1 (apoptosis and prognostic in breast cancer) COG3 (protein glycosylation/golgi function) CCNB1 (mitosis and prognosis in breast cancer) SRSF1 (RNA splicing and prognosis in small cell lung cancer) DIRAS3 (tumor suppressor gene) and LCK (resistance to apoptosis) (Fig. 3b). Hub proteins unique to rectal cancers were: IGF1R (proliferation, invasion, migration), TSC1 (cell growth) BRCA2 (DNA repair) and COPS5 (multiple pathways) (Fig. 3c).

BAP1 was found to have a prominent role in both RCC and LCC. Although there are several conserved interactions, the BAP1 interactome of LCC diverges from that of RCC. Among the conserved interacting proteins are: BRD4, ADAR, GAB2, SLC1A5, EIF4G1, ERCC5 and TP53BP1, BRD4, ADAR, MSH6, FOXM1 and XRCC5. Specific to LCC, BAP1 showed interactions with ERCC1, PRKCA, GATA6, JAK2, RAD51, TSC1, RSC1, NOTCH1, BCL2, KIT, PRKCD, CDH2, ARID1A, ASNS, SQSTM1 and DVL3. Specific to RCC, BAP1 was noted to interact with CDH1, MAPK14, MRE11A, MET, YAP1, STK11, ERBB3, PIK3CA, PXN, CHEK1, CTNNB1, STAT5A, EEF2K, G6PD, COG3, RBM15, BCL2A1, SYK, RELA and ANXA1.

Our results suggest BAP1 may have an essential role in carcinogenesis of colon cancer with conserved as well as divergent evolutionary interactions with other proteins in RCC and LCC that are largely absent in rectal cancers.

A somewhat surprising observation from this analysis is that the protein hubs and their interactomes are distinct for each of the anatomically defined tumor sites examined. Further, these protein signatures are not necessarily concordant with the somatic tumor profiles. Identifying alterations in tumor DNA and RNA have been of paramount importance. Clarifying post-transcriptional events and protein-protein interactions will also be highly relevant to understanding the variations in tumor biology and clinical behavior of these tumors. Prospective studies are needed to validate our findings and their implications in the clinical outcomes.

## Additional files

Additional file 1: Patients demographics from TCGA. (DOCX 19 kb)

Additional file 2: Inclusion and exclusion criteria for somatic mutation analysis. MSI-H, POLE mutation samples, rectosigmoid and transverse colon cancers were excluded  for analysis(highlighted green). (TIF 666 kb)

Additional file 3: Methods section. (DOCX 52.9 kb)

Additional file 4: **Tables S1-S3.** PicNiC statistics (bic and aic) for RCC, LCC and rectal cancers. (XLSX 43 kb)

Additional file 5: Somatic mutation analysis  for RCC, LCC and rectal cancers. (DOCX 433 kb)

Additional file 6: **Table S4.** Proteomics pathway and gene level analysis results. (XLSX 47 kb)

## Abbreviations

AIC: Akaike information criterion
BIC: Bayesian information criterion
CAPRI: Cancer progression inference algorithm
KDE: Kernel density estimator
LCC: Left colon cancer
MSKCC: Memorial sloan kettering cancer center
PiCnIc: Pipeline for cancer inference
PMID: Pubmed identifier
RCC: Right colon cancer
TCGA: The cancer genome atlas
TCPA: The cancer proteome atlas
