# Supplementary material for: Comparative proteogenomic analysis of right-sided colon cancer, left-sided colon cancer and rectal cancer reveals distinct mutational profiles
Source: Mol Cancer. 2018 Dec 21;17:177. doi: 10.1186/s12943-018-0923-9 (PMC6303985; doi:10.1186/s12943-018-0923-9)
Supplement: Supplementary file 1 — Patients demographics from TCGA. (DOCX 19 kb) [file 12943_2018_923_MOESM1_ESM.docx]

| Patients demographics from TCGA | | | | | |
| --- | --- | --- | --- | --- | --- |
| Patients Characteristics | Left colon cancer  n = 156 | Right colon cancer  n = 142 | Rectal cancer  n = 89 | Total  n = 387 | P-Value |
| Gender      Female      Male | 74 (47.4%) 82 (52.6%) | 63 (44.4%) 79 (55.6%) | 40 (44.9%) 49 (55.1%) | 177 (45.7%) 210 (54.3%) | 0.855 |
| Age | 64.8 ± 12.7 | 68.4 ± 12.0 | 66.7 ± 11.2 | 66.6 ± 12.2 | 0.040 |
| Race      American Indian OR Alaska native      Asian      African American      White      Unknown | 1 (0.6%) 3 (1.9%) 15 (9.6%) 70 (44.9%) 67 (42.9%) | 0 (0.0%) 4 (2.8%) 26 (18.3%) 74 (52.1%) 38 (26.8%) | 0 (0.0%) 1 (1.1%) 2 (2.2%) 24 (27.0%) 62 (69.7%) | 1 (0.3%) 8 (2.1%) 43 (11.1%) 168 (43.4%) 167 (43.2%) | < 0.001 |
| Histological diagnosis      Colon Adenocarcinoma      Colon Mucinous Adenocarcinoma      Rectal Adenocarcinoma      Rectal Mucinous Adenocarcinoma      Discrepancy      Data not Available | 138 (88.5%) 11 (7.1%) 4 (2.6%) 0 (0.0%) 1 (0.6%) 2 (1.3%) | 124 (87.3%) 18 (12.7%) 0 (0.0%) 0 (0.0%) 0 (0.0%) 0 (0.0%) | 0 (0.0%) 0 (0.0%) 80 (89.9%) 7 (7.9%) 0 (0.0%) 2 (2.2%) | 262 (67.7%) 29 (7.5%) 84 (21.7%) 7 (1.8%) 1 (0.3%) 4 (1.0%) | < 0.001 |
| Primary site      Ascending Colon      Cecum      Descending Colon      Hepatic Flexure      Rectum      Sigmoid Colon      Splenic Flexure | 0 (0.0%) 0 (0.0%) 17 (10.9%) 0 (0.0%) 0 (0.0%) 132 (84.6%) 7 (4.5%) | 53 (37.3%) 73 (51.4%) 0 (0.0%) 16 (11.3%) 0 (0.0%) 0 (0.0%) 0 (0.0%) | 0 (0.0%) 0 (0.0%) 0 (0.0%) 0 (0.0%) 89 (100.0%) 0 (0.0%) 0 (0.0%) | 53 (13.7%) 73 (18.9%) 17 (4.4%) 16 (4.1%) 89 (23.0%) 132 (34.1%) 7 (1.8%) | < 0.001 |
| AJCC pathological stage      Stage I      Stage II      Stage III      Stage IV      Discrepancy      Data not Available | 26 (16.7%) 53 (33.9%) 46 (29.5%) 29 (18.6%) 1 (0.6%) 1 (0.6%) | 23 (16.2%) 45 (31.7%) 44 (31%) 24 (16.9%) 2 (1.4%) 4 (2.8%) | 20 (22.5%) 28 (31.5%) 25 (28.1%) 15 (16.8%) 0 (0.0%) 1 (1.1%) | 69 (17.8%) 126 (32.5%) 115 (29.7%) 68 (17.5%) 3 (0.8%) 6 (1.6%) | 0.842 |
| MSSLH   MSI-L      MSS     Indeterminate | 32 (20.5%) 122 (78.2%)  2 (1.3%) | 27 (19.0%) 115 (81.0%)  0 (0.0%) | 11 (12.4%) 78 (87.6%)  0 (0.0%) | 70 (18.1%) 315 (81.4%)  2 (0.5%) | 0.216 |
| Continuous variables compared using one-way analysis of variance.  Categorical variables compared using chi-square test. | | | | | |
