# Supplementary figures and images for: Comparative proteogenomic analysis of right-sided colon cancer, left-sided colon cancer and rectal cancer reveals distinct mutational profiles
Source: Mol Cancer. 2018 Dec 21;17:177. doi: 10.1186/s12943-018-0923-9 (PMC6303985; doi:10.1186/s12943-018-0923-9)

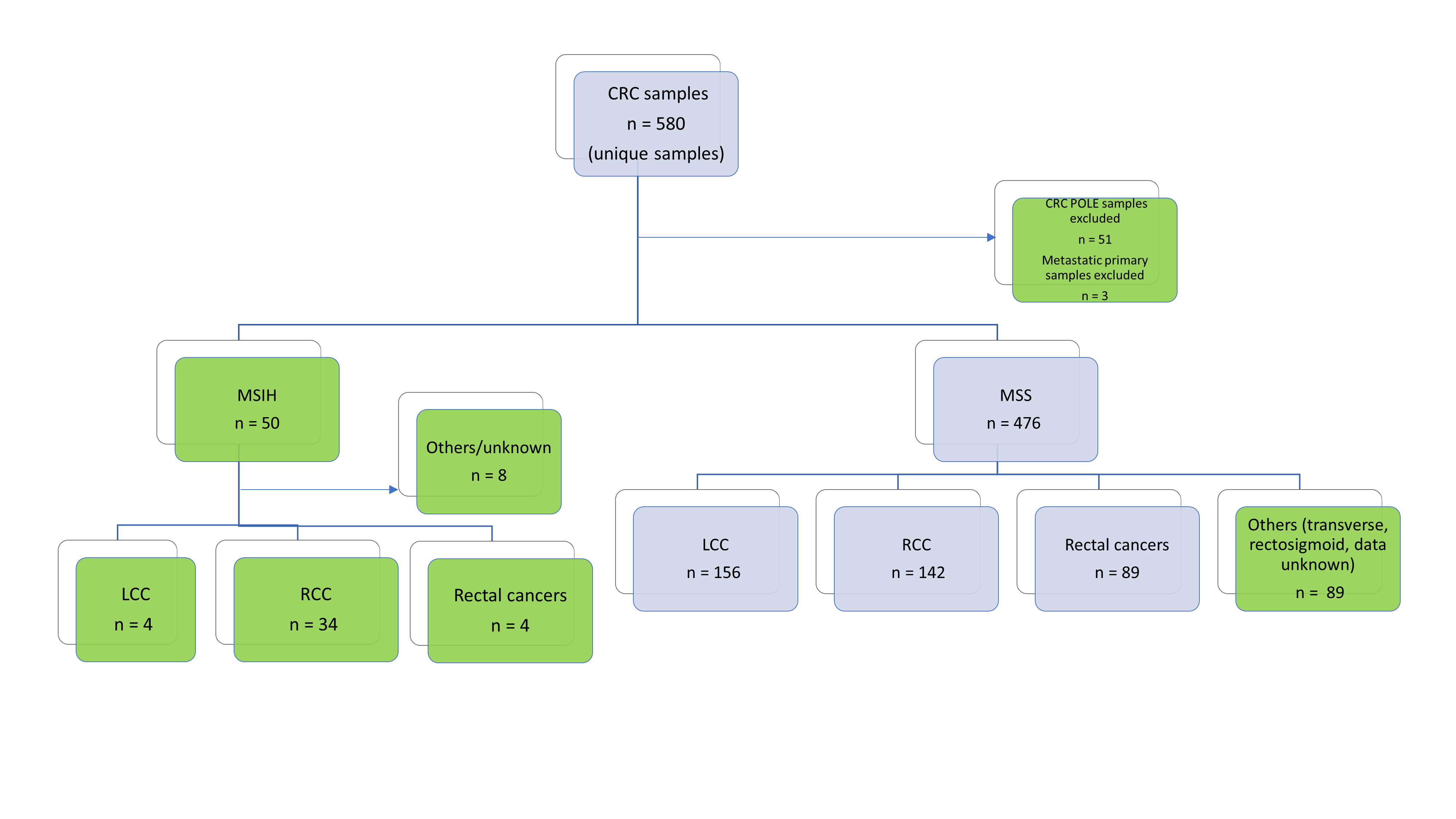

Supplement: Supplementary file 2 — Inclusion and exclusion criteria for somatic mutation analysis. MSI-H, POLE mutation samples, rectosigmoid and transverse colon cancers were excluded for analysis(highlighted green). (TIF 666 kb) [file 12943_2018_923_MOESM2_ESM.tif]
