# Supplementary material for: Comparative proteogenomic analysis of right-sided colon cancer, left-sided colon cancer and rectal cancer reveals distinct mutational profiles
Source: Mol Cancer. 2018 Dec 21;17:177. doi: 10.1186/s12943-018-0923-9 (PMC6303985; doi:10.1186/s12943-018-0923-9)
Supplement: Supplementary file 3 — Methods section. (DOCX 52.9 kb) [file 12943_2018_923_MOESM3_ESM.docx]

**Methods**

**Pipeline for Cancer Inference (PiCnIc) for evolutionary trajectories**

Somatic mutation data was obtained GDC Legacy Archive TCGA provisional colorectal cancer data set in the form of MAF files on January 23, [1] (https://gdc-portal.nci.nih.gov/legacy-archive/files/3437ecf9-355d-4d35-afb4-ffe1a705c206; https://gdc-portal.nci.nih.gov/legacy-archive/files/7c3e8456-c39f-4292-8cc2-374b14c75446). Copy number data for the colorectal cancer dataset was downloaded from CBioportal January 30, 2018 [2,3]. Available copy number data were matched to available somatic mutation data based on location of the primary tumor: right-sided colon cancer (RCC; (n=135), left sided colon cancer (LCC; n=143), rectal cancers (n=76).

We applied the Pipeline for Cancer Inference (PiCnIc) algorithm using default parameters provided by the authors to determine evolutionary trajectories of cross-sectional TCGA data (https://github.com/BIMIB-DISCo/PiCnIc-COADREAD/tree/master/scripts) [4]. We examined a total of 50 known driver genes: 33 genes that were selected by Muzny et al. in their original report on colorectal cancer [5] and 17 unique genes from a pan-cancer analysis by Ciriello et al. that are ubiquitous driver alterations in solid tumors [6]. As previously described by Caravagna et al [4], exclusivity among genes in the WNT (*APC* and *CTNNB1*) and RAF (*KRAS*, *NRAS*, and *BRAF*) pathways were also used as a parameters in our analysis. We ran CAPRI as the last step of PiCnIc. For statistical analysis, we used the Mann-Withney *U* test with statistical significance 0.05, after 100 nonparametric bootstrap iterations. We considered the Akaike information criterion (AIC) score of 40% and the Bayesian information criterion (BIC) score of 30% significant. Nonparametric and statistical bootstrap estimations are shown in Additional File 4: Tables S1-S3

**Mutation Hotspot Analysis**

Mutation data was downloaded from the GDC Legacy Archive TCGA provisional colorectal cancer data set in the form of MAF files on January 23, 2018(1). Samples were parsed into RCC (n=142), LCC (n=156), and rectal cancers (n=89) based on location of primary tumor. Hypermutated samples and samples containing POLE mutations were removed from analysis. Hotspot analysis was carried out on the remaining samples using an algorithm previously described by Chang et al. [7]. Publicly available R scripts were applied to our data with default parameters. Lollipop plots and somatic mutation plots were created using MAF tools [8].

**Somatic Mutation Analysis**

We conducted somatic mutation analysis of RCC, LCC and rectal cancers (See Additional File 5). Similar to the hotspot analysis, somatic mutation data was downloaded from the GDC Legacy Archive provisional data set for colon and rectal adenocarcinoma on January 23, 2018 and parsed based on location (RCC n=142, LCC n=156, rectal cancers n=89). We then parsed the data based on location and analyzed it using the ConsensusDriver algorithm to highlight potential Driver Mutations [9]. Consensus Driver is a relatively new driver prediction algorithm that integrates previous driver prediction algorithms (fathmm, CHASM, OncoIMPACT, DriverNet, MutSigCV, OncodriveFM) and significantly improves the quality of predictions and discovery of novel significantly mutated genes. MAF files were parsed into individual sample files and converted to vcf files using MAF2VCF perl script (https://github.com/mskcc/vcf2maf) and passed through the ConsensusDriver Algorithm using colon adenocarcinoma and rectum adenocarcinoma cancer subtype options as described by Bertrand et al [9]. The resulting genes were combined into a list. Known false positive driver mutations as previously identified by Bertrand et al. were removed from our analysis. Frequencies of these mutated gene were compared in each specified location. Genes with mutational frequencies less than 5% were excluded from our study. The remaining genes were compared among each location and evaluated for enrichment (≥5% frequency of mutation and p<0.05).

**Proteogenomic analysis**

Proteomics analysis was performed by using our previously described algorithm [10]. Reverse Phase Protein Array (RPPA) samples (COAD and READ) were downloaded from The Cancer Proteome Atlas (TCPA) website (http://tcpaportal.org/tcpa/) on January 23, 2018 [11]. The samples included 98 RCC, 109 LCC and 67 rectal cancer samples.

As shown in Additional File 6: Table S4, the rankings of the association estimators identified by the average performance score varied according to the constructed module sizes and the selected groups. In the pathway-level analysis, the best performing methods based on the selected groups were MM for RCC and KDE for LCC and rectal cancers. Similarly, in the gene-level analysis, best performing methods based on the selected groups were MM for RCC and KDE for LCC and rectal cancers.

The sub networks of the module hub genes identified by the association estimators, which provided the highest precision scores based on a statistical test (with a p<0.05), were generated by Cytoscape [12] for all selected groups. These genes were then compared to those registered in DisGeNET which contains experimentally confirmed associations with listed diseases [13].

**Abbreviations**

AIC – Akaike Information Criterion

BIC – Bayesian Information Criterion

CAPRI – Cancer Progression Inference Algorithm

COAD – Colon Adenocarcinoma

GDC – Genomic Data Commons

INDEL – Insertion or Deletion mutations

KDE – Kernel Density Estimator

LCC – Left Colon Cancer

MM – Miller Madow association estimator

PiCnIC – Pipeline for Cancer Inference

RCC – Right Colon Cancer

READ – Rectum Adenocarcinoma

RPPA – Reverse Phase Protein Array

SNV – Single Nucleotide Variant

TCGA – The Cancer Genome Atlas

TCPA – The Cancer Proteome Atlas

**References:**

1. Grossman RL, Heath AP, Ferretti V, Varmus HE, Lowy DR, Kibbe WA, et al. Toward a Shared Vision for Cancer Genomic Data. N Engl J Med. 2016;375:1109–12.

2. Cerami E, Gao J, Dogrusoz U, Gross BE, Sumer SO, Aksoy BA, et al. The cBio Cancer Genomics Portal: An Open Platform for Exploring Multidimensional Cancer Genomics Data. Cancer Discov. 2012;2:401 LP-404.

3. Gao J, Aksoy BA, Dogrusoz U, Dresdner G, Gross B, Sumer SO, et al. Integrative Analysis of Complex Cancer Genomics and Clinical Profiles Using the cBioPortal. Sci Signal. 2013;6:pl1-pl1.

4. Caravagna G, Graudenzi A, Ramazzotti D, Sanz-Pamplona R, De Sano L, Mauri G, et al. Algorithmic methods to infer the evolutionary trajectories in cancer progression. Proc Natl Acad Sci. 2016;113:E4025–34.

5. Muzny DM, Bainbridge MN, Chang K, Dinh HH, Drummond JA, Fowler G, et al. Comprehensive molecular characterization of human colon and rectal cancer. Nature . Nature Publishing Group; 2012;487:330–7.

6. Ciriello G, Miller ML, Aksoy BA, Senbabaoglu Y, Schultz N, Sander C. Emerging landscape of oncogenic signatures across human cancers. Nat Genet. Nature Publishing Group; 2013;45:1127–33.

7. Chang MT, Asthana S, Gao SP, Lee BH, Chapman JS, Kandoth C, et al. Identifying recurrent mutations in cancer reveals widespread lineage diversity and mutational specificity. Nat Biotechnol. 2016;34:155–63.

8. Mayakonda A, Lin D, Assenov Y, Plass C, Koeffler PH (2018). “Maftools: efficient and comprehensive analysis of somatic variants in cancer.” Genome Research. doi: [10.1101/gr.239244.118](http://doi.org/10.1101/gr.239244.118)

9. Bertrand D, Drissler S, Chia B, Koh JY, Li C, Suphavilai C, et al. ConsensusDriver Improves Upon Individual Algorithms For Predicting Driver Alterations In Different Cancer Types And Individual Patients — A Toolbox For Precision Oncology. bioRxiv . 2017.

10. Erdoğan C, Kurt Z, Diri B. Estimation of the proteomic cancer co-expression sub networks by using association estimators. Yamanishi Y, editor. PLoS One. 2017;12:e0188016.

11. Li J, Lu Y, Akbani R, Ju Z, Roebuck PL, Liu W, et al. TCPA: a resource for cancer functional proteomics data. Nat Methods. 2013;10:1046–7.

12. Shannon P, Markiel A, Owen Ozier 2, Baliga NS, Wang JT, Ramage D, et al. Cytoscape: a software environment for integrated models of biomolecular interaction networks. Genome Res. 2003;2498–504.

13 Piñero J, Queralt-Rosinach N, Bravo À, Deu-Pons J, Bauer-Mehren A, Baron M, et al. DisGeNET: A discovery platform for the dynamical exploration of human diseases and their genes. Database. 2015;2015:1–17.
