# Supplementary material for: Comparative proteogenomic analysis of right-sided colon cancer, left-sided colon cancer and rectal cancer reveals distinct mutational profiles
Source: Mol Cancer. 2018 Dec 21;17:177. doi: 10.1186/s12943-018-0923-9 (PMC6303985; doi:10.1186/s12943-018-0923-9)
Supplement: Supplementary file 5 — Somatic mutation analysis for RCC, LCC and rectal cancers. (DOCX 433 kb) [file 12943_2018_923_MOESM5_ESM.docx]

**Somatic Mutation Analysis**

The ConsensusDriver algorithm identified 25 significantly mutated genes (≥5% of tumors) in all three tumor locations, including mutations in the WNT, P53 and TGFB pathways, in agreement with other studies [1–9]. In addition, we identified several novel driver mutations within the DNAH8, DST, PAPPA, and TNR genes (Supplemental Figure 1).

Comparison of significantly mutated genes (SMGs) between RCC, LCC and rectal cancers identified 9 SMGs that were significantly enriched (≥5% of tumor samples, p<0.05) in RCC (Supplemental Figure 2a-c). These included RTK/RAS pathway genes: KRAS and BRAF; IGF/PI3K pathway gene: PIK3CA; WNT pathway gene: SOX9; TGFB pathway gene: ACVR2A; and MYC-pathway gene: EP400. Novel genes significantly enriched include: FRY, FLNA and BCOR. FLNA and BCOR are associated with tumor progression and metastasis [10] whereas FRY encodes a microtubule-binding protein which is conserved across species [11].

We also discovered somatic alterations in genes likely associated with tumor invasiveness and progression that are enriched in RCC compared to LCC (all p<0.04) but not rectal cancers (all p>0.05). These genes include RELN [12], MAP2 [13], NCAM1 [14] and RUNX1T1 [15,16]. This discrepancy may be due to sample size difference (RCC n=142, rectal cancer n=89). Altogether, these genes are mutated in 37% of RCC (52/142) versus 10% of LCC (15/156) and showed a tendency towards mutual exclusivity. TRRAP was also significantly enriched in RCC (6%) compared to LCC (1%, p<0.02) but not rectal cancers (2%, p=0.2). Recently, TRRAP was found to be essential for regulating p53 mutant levels in lymphomas by preventing its degradation [17]. Given its enrichment in RCC, it may play a potential role to that effect in colorectal cancer.

Conversely, only one SMG was enriched in LCC, PCDH10. This gene was statistically enriched compared to rectal cancer (7% vs. 1%, p=0.03) but was not statistically enriched compared to RCC (7% vs. 4%, p=0.18). This gene is a tumor suppressor gene shown to be an independent predictor of colorectal metastasis [18].

Compared to LCC, rectal cancers were enriched for LRP1B (20% vs. 11%, p=0.04). LRP1B functions as a LDL receptor-related protein involved in promoting growth and migration in colorectal cancers [19]. However, LRP1B did not reach statistical significance when comparing LCC to RCC (20% vs. 16%, p=0.43). Similarly, ERBB2 somatic mutations were significantly enriched in rectal cancers compared to LCC (6% vs. 1%, p=0.01) but not RCC (6% vs. 2%, p=0.15).

In addition, we also analyzed the genomic differences between RCC, LCC and rectal cancer among a cohort of patients with metastatic colorectal cancer (represented by the MSKCC cohort) [20]. Mutational analyses were done with the 468 gene MSK-IMPACT clinical sequencing platform. Several right sided enriched genes are not in the MSKCC gene panel and thus were not included in the analysis. These genes include: FRY, NCAM1, RELN, CDH22, ACVR2A, SMARCA2, TRRAP, MXRA5, EP400, FLNA, MAP2 and RUNX1T1.  As in the early stage tumors from the TCGA cohort, KRAS and PIK3CA mutations were enriched in metastatic RCC compared to LCC and rectal cancers (all p<0.005) suggesting that they may have a larger role to play in RCC development. Similarly, BRAF was highly enriched in RCC compared to LCC and rectal cancers in both data sets (all p<0.04). Interestingly, ZFHX3, mutated at low rates in the TCGA cohort was significantly enriched in metastatic RCC (5%) compared to metastatic LCC (2%) and metastatic rectal cancers (0%; all p<0.03; Supplemental Figure 2a-c).

TP53 was more highly mutated in LCC (82%) and rectal cancers (84%) than RCC (67%) in the MSKCC cohort (both p<0.001). This trend was also seen in the TCGA cohort, though not to statistical significance (71% LCC vs. 61% RCC, p=0.07; 71% rectal cancer vs. 61% RCC, p=0.14). LCC was also highly enriched for APC mutations compared to RCC in the advanced stage colorectal cancer cohort (82% vs. 71%, p=0.002), whereas APC mutations were equally distributed between RCC and LCC in the TCGA data set (78% vs. 77%, respectively). FBXW7 mutations were enriched in rectal cancers (16%) compared to either LCC or RCC (5% and 7% respectively, both p<0.01; Supplemental Figure 2a-c).

Next, we analyzed the concordance or discordance of driver mutations between biopsies taken from primary tumor sites vs. metastatic sites within the MSKCC colorectal cancer cohort. We found minimal differences in driver mutations between the primary sites and metastatic sites. Only 5 genes, mostly among RCC made our statistical cut off for enrichment (≥5%, p < 0.05). In RCC, primary tumor biopsies were enriched in ERBB2 and ARID1A (11% vs. 1%, p = 0.002; 8% vs. 1%, p = 0.01, respectively), whereas metastatic site biopsies had higher enrichment for NRAS and EPHA5 (6% vs. 0%, p = 0.02; 9% vs 2% p = 0.02, respectively). In rectal cancers, FBXW7 was enriched in primary site biopsies compared to metastatic sites (5% vs 0%, p = 0.03).  Surprisingly, no differences were seen in driver mutation enrichment in LCC when comparing primary site biopsies to metastatic site biopsies.

Although AMER1 not identified as a significantly mutated gene by ConsensusDriver, we analyzed the distribution of AMER1 mutations in colorectal cancers as several studies have previously identified such mutations in colorectal cancer [21,22]. We found this gene to be significantly enriched in RCC compared to LCC and rectal cancer in the TCGA data set (both 23% vs. 3%, p<0.0001). Mutations in AMER1 and other keys genes of the β-catenin destruction complex were mutually exclusive of each other (Supplementary Figure 3). However, these findings were not replicated in the MSKCC data set.


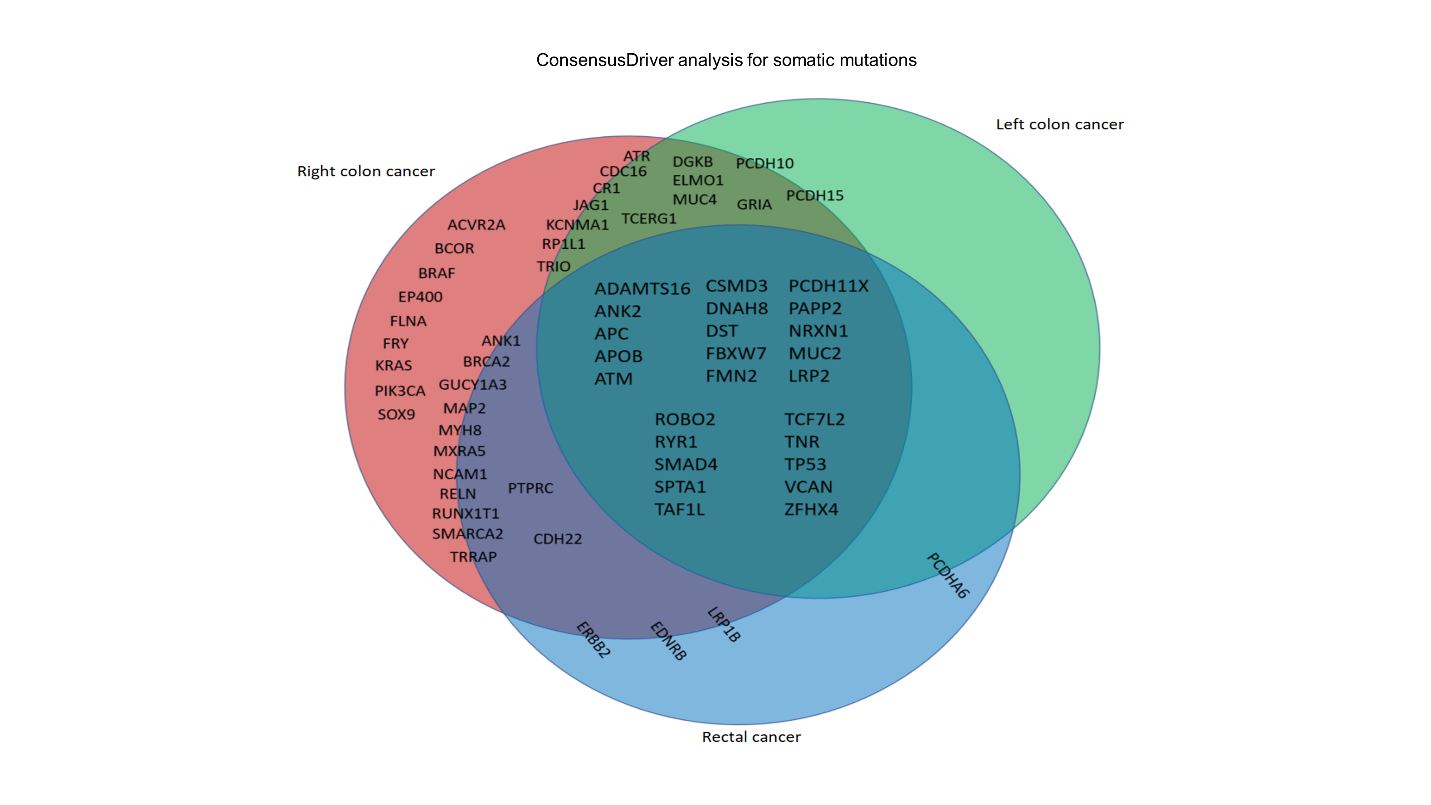


Supplemental Figure 1 shows ConsensusDriver analysis for somatic mutation enrichment. Genes located within each area met criteria for statistically significant mutation enrichment (frequency ≥ 5%, chi-square p < 0.05; RCC n=142, LCC n=156, Rectal cancers n=89) compared to the other two anatomical locations (e.g. KRAS mutations were found statistically enriched in right colon cancers compared to left colon cancers and rectal cancers). Genes located on the borders of the Venn diagram had enrichment for mutations in both locations but only met statistical significance for enrichment to one site but not both (e.g. ERBB2 mutations were enriched in rectal cancers compared to left colon cancers with statistical significance but not to right colon cancers. They were enriched in right colon cancers compared to left colon cancers but not to statistical significance).


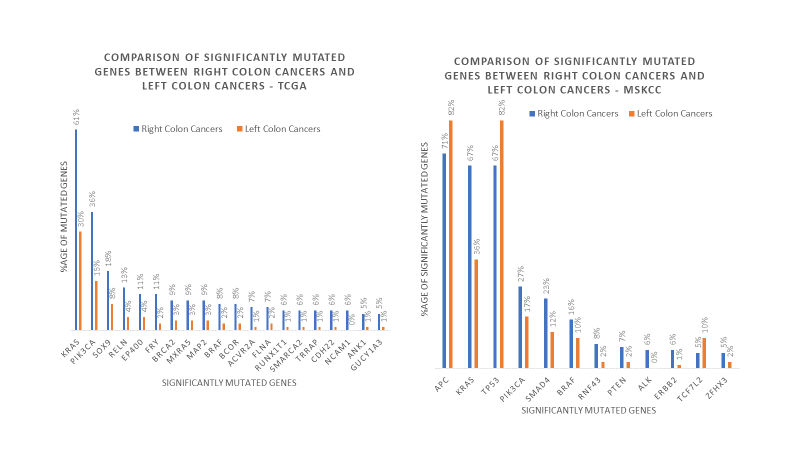


Supplemental Figure 2a shows significantly mutated genes (SMGs) between TCGA and MSKCC data set for right-sided colon cancers and left-sided colon cancers. SMGs were defined as presence of genes in ≥ 5% of tumor samples with p < 0.05. Twenty SMGs were enriched in RCC compared to LCC in TCGA and twelve SMGs were enriched in MSKCC. Three SMGs (KRAS, PIK3CA, BRAF) were common between the two data sets that were enriched in RCC.


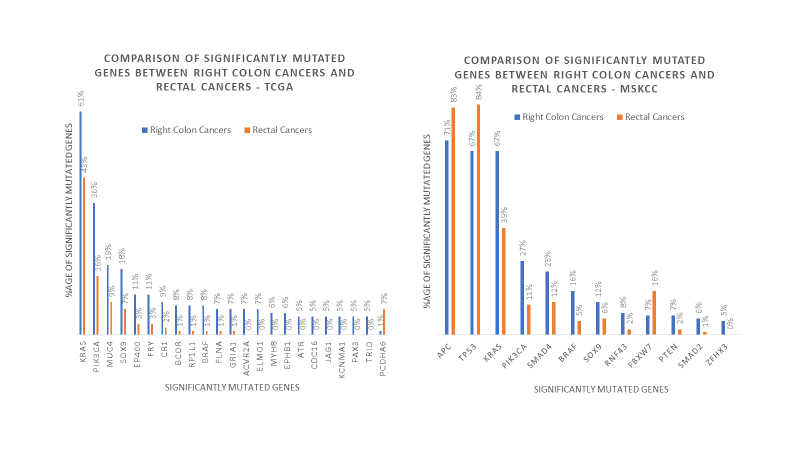


Supplemental Figure 2b shows the SMGs between TCGA and MSKCC data set for right-sided colon cancers and rectal cancers. Twenty-three SMGs were enriched in RCC vs. rectal cancers in TCGA and 12 SMGs in MSKCC. Four SMGs (KRAS, PIK3CA, SOX9, BRAF) were common between the TCGA and MSKCC that were enriched in RCC.


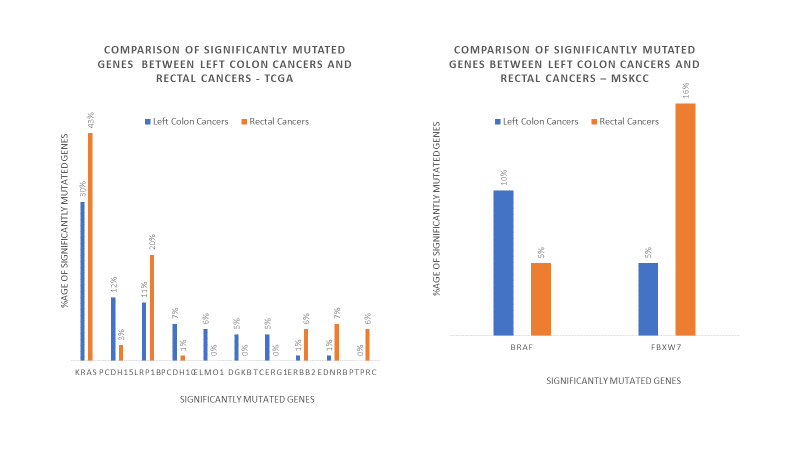


Supplemental Figure 2c shows the SMGs between TCGA and MSKCC data set for LCC and rectal cancers. 10 SMGs were identified in TCGA and 2 SMG’s were identified in MSKCC data set. No similar SMGs were identified between the two data sets.


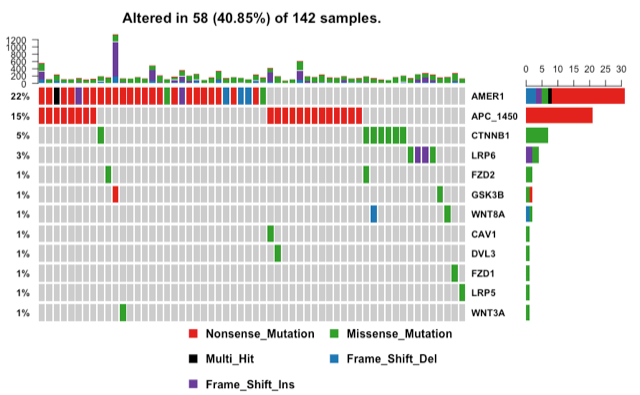


Supplemental Figure 3: Oncoplot of right colon cancer (RCC) samples. APC_1450 represents the R1450* mutation found in RCC. Both AMER1 and APC R1450* tend toward mutual exclusivity with WNT pathway genes.

**Abbreviations**

CRC – Colorectal cancer

MSKCC – Memorial Sloan Kettering Cancer Center

MSK-IMPACT – Memorial Sloan Kettering Cancer Center 468-gene oncopanel for targeted sequencing

LCC – Left colon cancer

RCC – Right colon cancer

SMG – Significantly Mutated Gene

TCGA – The Cancer Genome Atlas

**References**

1. Hu W, Yang Y, Li X, Huang M, Xu F, Ge W, et al. Multi-omics Approach Reveals Distinct Differences in Left- and Right-sided Colon Cancer. Mol Cancer Res. 2017;molcanres.0483.2017.

2. Schell MJ, Yang M, Teer JK, Lo FY, Madan A, Coppola D, et al. A multigene mutation classification of 468 colorectal cancers reveals a prognostic role for APC. Nat Commun . Nature Publishing Group; 2016;7:1–12.

3. Lipsyc M, Yaeger R. Impact of somatic mutations on patterns of metastasis in colorectal cancer. J. Gastrointest. Oncol. 2015. p. 645–9.

4. Haan JC, Labots M, Rausch C, Koopman M, Tol J, Mekenkamp LJM, et al. Genomic landscape of metastatic colorectal cancer. Nat Commun. Nature Publishing Group; 2014;5:1–12.

5. Yaeger R, Chatila WK, Lipsyc MD, Hechtman JF, Cercek A, Sanchez-Vega F, et al. Clinical Sequencing Defines the Genomic Landscape of Metastatic Colorectal Cancer. Cancer Cell. Elsevier Inc.; 2018;33:125–136.e3.

6. Borras E, Lucas FAS, Chang K, Zhou R, Masand G, Fowler J, et al. Genomic landscape of colorectal mucosa and adenomas. Cancer Prev Res. 2016;9:417–27.

7. Yu J, Wu WKK, Li X, He J, Li X-X, Ng SSM, et al. Novel recurrently mutated genes and a prognostic mutation signature in colorectal cancer. Gut. 2015;64:636–45.

8. Muzny DM, Bainbridge MN, Chang K, Dinh HH, Drummond JA, Fowler G, et al. Comprehensive molecular characterization of human colon and rectal cancer. Nature . Nature Publishing Group; 2012;487:330–7.

9. Druliner BR, Wang P, Bae T, Baheti S, Slettedahl S, Mahoney D, et al. Molecular characterization of colorectal adenomas with and without malignancy reveals distinguishing genome, transcriptome and methylome alterations. Sci Rep. 2018;8:3161.

10. Tian Z-Q, Shi J-W, Wang X-R, Li Z, Wang G-Y. New cancer suppressor gene for colorectal adenocarcinoma: Filamin A. World J Gastroenterol. 2015;21:2199–205.

11. Nagai T, Mizuno K. Multifaceted roles of Furry proteins in invertebrates and vertebrates. J Biochem. 2014;155:137–46.

12. Castellano E, Molina-Arcas M, Krygowska AA, East P, Warne P, Nicol A, et al. RAS signalling through PI3-Kinase controls cell migration via modulation of Reelin expression. Nat Commun. 2016;7:11245.

13. Soltani MH, Pichardo R, Song Z, Sangha N, Camacho F, Satyamoorthy K, et al. Microtubule-associated protein 2, a marker of neuronal differentiation, induces mitotic defects, inhibits growth of melanoma cells, and predicts metastatic potential of cutaneous melanoma. Am J Pathol. 2005;166:1841–50.

14. Fernández-Briera A, García-Parceiro I, Cuevas E, Gil-Martín E. Effect of human colorectal carcinogenesis on the neural cell adhesion molecule expression and polysialylation. Oncology. 2010;78:196–204.

15. Alfayez M, Vishnubalaji R, Alajez NM. Runt-related Transcription Factor 1 (RUNX1T1) Suppresses Colorectal Cancer Cells Through Regulation of Cell Proliferation and Chemotherapeutic Drug Resistance. Anticancer Res. 2016;36:5257–63.

16. Nasir A, Helm J, Turner L, Chen D-T, Strosberg J, Hafez N, et al. RUNX1T1. Pancreas. 2011;40:627–33.

17. Jethwa A, Słabicki M, Hüllein J, Jentzsch M, Dalal V, Rabe S, et al. TRRAP is essential for regulating the accumulation of mutant and wild-type p53 in lymphoma. Blood. 2018;

18. Ying J, Li H, Seng TJ, Langford C, Srivastava G, Tsao SW, et al. Functional epigenetics identifies a protocadherin PCDH10 as a candidate tumor suppressor for nasopharyngeal, esophageal and multiple other carcinomas with frequent methylation. Oncogene. 2006;25:1070–80.

19. Wang Z, Sun P, Gao C, Chen J, Li J, Chen Z, et al. Down-regulation of LRP1B in colon cancer promoted the growth and migration of cancer cells. Exp Cell Res. 2017;357:1–8.

20. Yaeger R, Chatila WK, Lipsyc MD, Hechtman JF, Cercek A, Sanchez-Vega F, et al. Clinical Sequencing Defines the Genomic Landscape of Metastatic Colorectal Cancer. Cancer Cell. Elsevier Inc.; 2018;33:125–136.e3.

21. Sanz-Pamplona R, Lopez-Doriga A, Paré-Brunet L, Lázaro K, Bellido F, Alonso MH, et al. Exome sequencing reveals AMER1 as a frequently mutated gene in colorectal cancer. Clin Cancer Res. 2015;21:4709–18.

22. Muzny DM, Bainbridge MN, Chang K, Dinh HH, Drummond JA, Fowler G, et al. Comprehensive molecular characterization of human colon and rectal cancer. Nature. Nature Publishing Group; 2012;487:330–7.
